# Liver resection for young patients with large hepatocellular carcinoma: a single center experience from China

**DOI:** 10.1186/1477-7819-12-175

**Published:** 2014-06-03

**Authors:** Xi-yu Liu, Jiang-feng Xu

**Affiliations:** 1 Department of surgery, Yiwu Affiliated Hospital of Zhejiang University School of Medicine, east building in huajiachi campus,kaixuan road 268, 310020 Hangzhou, Zhejiang, China

**Keywords:** Liver resection, Young patients, Large hepatocellular carcinoma

## Abstract

**Background:**

To investigate retrospectively the clinicopathological characteristics and outcomes of young patients with large hepatocellular carcinoma after hepatectomy.

**Methods:**

From January 2003 to December 2012, a total of 153 patients with large hepatocellular carcinoma (HCC) who received liver resection were included in the study. The clinicopathological features were analyzed retrospectively. The perioperative data were compared between those aged <40 years (the young group) and those aged >40 years (the older group). Prognostic factors and long-term survival were evaluated.

**Results:**

The young group had more hepatitis B virus-related HCC than the older group (87.2% vs 66.3%, *P* = 0.031). In the young group, 15 patients (21.5%) were overweight (body mass index 25 to 29.9 kg/m^2^) or obese (body mass index ≥30 kg/m^2^), and 38 patients (45.8%) were overweight or obese in the older group (*P* = 0.032). Other clinicopathological characteristics were similar between the two groups. The perioperative data showed that the older group had more pulmonary infection after hepatectomy. Vascular invasion and high Edmondson-Steiner grade were the independent prognostic factors for long-term survival. There was no statistical difference between the young group and the older group in overall survival and disease-free survival (*P* = 0.109 and *P* = 0.087, respectively).

**Conclusions:**

Liver resection for young patients with large HCC was safe and efficacious and should be recommended.

## Background

Hepatocellular carcinoma (HCC) is one of the most common malignancies worldwide, especially in China. HCC ranks second as a cause of cancer death overall in China [1-4]. Many studies have reported that hepatectomy could be performed satisfactorily for large HCC (>5 cm) with acceptable mortality [5-7]. However, there are few reports about the clinicopathological features and outcomes in young patients with large HCC after hepatectomy, and the significance of hepatectomy for these patients therefore remains unknown.

In USA and Europe, which are not hepatitis B endemic areas, patients younger than 40 years of age with large HCC are reported to be rare;however, young patients with large HCC are not uncommon in China. To investigate the clinicopathological features as well as the long-term outcomes after hepatectomy of young patients with large HCC, we performed a retrospective study of patients with large HCC undergoing hepatectomy whose ages were younger than 40 years (the young group), compared to those patients aged above 40 years (the older group).

## Methods

From January 2003 to December 2012, 153 patients with large HCC (>5 cm) were treated surgically in the Affiliated Hospital of Zhejiang University School of Medicine. Patients younger than 40 years of age (n = 70) were defined as the young group And patients aged above 40 years (n = 83) were defined as the older group. Preoperative evaluation protocol included blood biochemistry, chest radiography, liver and renal function tests, ultrasonography, contrast computed tomography and indocyanine green clearance test.

Liver resection was undertaken in the patients with good cardiopulmonary and renal function, Pugh–Child’s grades A and B, and indocyanine green test at 15 min <15%.

All intraoperative and postoperative complications were reviewed retrospectively through medical records. Complications (Clavien-Dindo gradesI-V) contained ascites, wound infection, pleural effusion, pulmonary infection, biliary fistula, liver failure and bleeding. Follow-up data were obtained by direct communication with patients after they underwent hepatic resection. All patients were examined for recurrence by clinical examination, alpha-fetoprotein (AFP) and ultrasonography. The follow-up period was calculated from the date of surgery to the date of either death or last follow-up. Prior informed consent was obtained from all patients and the study was approved by the Ethics Committee of Yiwu affiliated hospital of zhejiang university school of medicine.

Continuous variables were expressed as mean ± SD and compared using the independent-samples *t* test. Categorical data analysis used the rank test or chi-square test. Survival analysis, including overall survival and disease-free survival, was estimated by the Kaplan-Meier survival method and compared using the log-rank test. Univariate and multivariate analysis by the Cox proportional hazard regression model was used to identify independent prognostic factors. All statistical analyses were performed using statistical software (SPSS 13.0 for Windows;SPSS, Chicago, IL, USA). *P* < 0.05 was considered to be statistically significant.

## Results

### Clinicopathologic features of patients with large hepatocellular carcinoma

The clinicopathologic parameters of the 153 patients with large HCC who underwent liver resection are shown in Table 1. The age of the young group was 32 ± 5 years compared to 55 ± 9 years in the older group. In the young group, 61 patients (87.2%) had positive hepatitis B in serologic test results; however, only 55 patients (66.3%) had positive hepatitis B in the older group. In the young group, 15 patients (21.4%) were overweight (body mass index (BMI) 25 to 29.9 kg/m^2^) or obese (BMI ≥30 kg/m^2^), and 38 patients (45.8%) were overweight or obese in the older group (*P* = 0.032). Clinicopathologic characteristics, including size of tumor, vascular invasion, tumor number, capsular formation, AFP level, liver cirrhosis, and Child-Pugh classification, showed no statistical difference between two groups.

**Table 1:** Clinicopathologic features of 153 patients with large hepatocellular carcinoma

**Variables**	**Mean ± SD or number (%) of patients**		
	**Young group (n = 70)**	**Older group (n = 83)**	***P *** **value**
Gender			
Female	17 (24.2%)	11(13.3%)	0.196
Male	53 (75.8%)	72(86.7%)	
Age (years)	32 ± 5	55 ± 9	**<0.001**
Hepatitis B status			
Negative	9 (12.8%)	28(33.7%)	**0.031**
Positive	61 (87.2%)	55(66.3%)	
Capsular formation			
Presence	32 (45.7%)	29(34.9%)	0.535
Absence	38 (54.3%)	54(65.1%)	
Tumor number			
Single	49(70.0%)	60(72.3%)	0.654
Multiple	21(30.0%)	23(27.7%)	
AFP level			
Negative	21 (30.0%)	32(38.5%)	0.616
Positive	49 (70.0%)	51(61.5%)	
Liver cirrhosis			
Absent	38 (54.3%)	44(53.0%)	0.851
Present	32 (45.7%)	39(47.0%)	
Child-Pugh classification			
A	56 (80.0%)	73(87.9%)	0.762
B	14 (20.0%)	10(12.1%)	
Tumor size (cm)	7.3 ± 2.1	7.9 ± 2.7	0.801
Vascular invasion			
Absent	37 (52.8%)	49(59.0%)	0.837
Present	33(47.2%)	34(41.0%)	
BMI			
Normal weight	55(78.6%)	45(54.2%)	**0.032**
Overweight/obese	15(21.4%)	38(45.8%)	

*P* values in bold indicate statistical significance between groups. AFP, alpha-fetoprotein; BMI, body mass index.

### Perioperative data

The intraoperative and postoperative data of 153 patients with large HCC who underwent liver resection are shown in Table 2. In the young group, non-anatomical resection was used in 31 (44.3%) patients, and 39 patients (55.7%) patients had hemihepatectomy or extended hemihepatectomy. In the young group, the surgical resection margin was ≤1 cm in 34 (48.6%) patients compared to 39 (47%) patients in the older group. The time for hepatic resection was 188 ± 9 minutes in the young group compared to 193 ± 23 minutes in the older group. The volume of blood loss was 1,196 ± 638 ml in the young group with 36 (51.4%) patients losing <1,000 ml compared to 1,226 ± 768 in the older group with 39 (47%) patients losing <1,000 ml. In the young group, 32 (45.7%) patients had no blood transfusion, the length of hospital stay was 14 ± 5 days with no hospital death, and the overall postoperative complication rate was 23% (16 patients). In the older group, 30 (36.1%) patients had no blood transfusion, the length of hospital stay was 16 ± 7 days, and the overall postoperative complication rate was 22.9% (19 patients).

**Table 2:** Perioperative data

**Variables**	**Mean ± SD or number (%) of patients**		
	**Young group (n = 70)**	**Older group (n = 83)**	***P *** **value**
Type of surgical resection			
Non-anatomical resection	31(44.3%)	36(43.4%)	0.756
Hemihepatectomy/extended hemihepatectomy	39(55.7%)	47(56.6%)	
Surgical resection margin (cm)			
≤1	34(48.6%)	39(47%)	0.936
>1	36(51.4%)	44(53%)	
Operative time (minutes)	188 ± 9	193 ± 23	0.673
Time for inflow occlusion (minutes)	13 ± 11	15 ± 13	0.516
Blood loss (ml)	1,196 ± 638	1,226 ± 768	0.475
<1,000	36 (51.4%)	39(47%)	0.561
≥1,000	34 (48.6%)	44(53%)	
Blood transfusion (patients)			
With	38 (54.3%)	53(63.9%)	0.625
Without	32(45.7%)	30(36.1%)	
Hospital mortality	0	1(1.2%)	0.543
Complications	16 (23.0%)	19(22.9%)	0.928
Hospital stay (days)	14 ± 5	16 ± 7	0.376

### Postoperative complications

There was no significant difference in the overall postoperative complication rate between the two groups (23% vs 22.9%, *P* = 0.928; Table 3). The common complications of the two groups were ascites, wound infection, pleural effusion, pulmonary infection, biliary fistula, liver failure and bleeding. The most common complication in the young group was bleeding (8.6%), and the most common complication in the older group was pulmonary infection (13.3%). Pulmonary infection showed a significant difference between the two groups (*P* = 0.041). The only postoperative death was caused by liver failure in the older group (Table 3).

**Table 3:** Postoperative complications

	**Number (%) of patients**		
**Complications**	**Young group**	**Older group**	***P *** **value**
Overall	16 (23.0%)	19 (22.9%)	0.928
Ascites	2 (2.8%)	1 (1.2%)	0.326
Wound infection	2(2.8%)	2(2.4%)	0.657
Pleural effusion	1(1.4%)	1(1.2%)	0.536
Pulmonary infection	1(1.4%)	11(13.3%)	**0.041**
Biliary fistula	2 (2.8%)	1(1.2%)	0.326
Liver failure	2(2.8%)	1(1.2%)*	0.326
Bleeding	6(8.6%)	2(2.4%)	0.084

*Caused postoperative death. *P* values shown in bold indicate statistical significance between groups.

### Long-term survival and prognostic factors of patients with large hepatocellular carcinoma after hepatectomy

**Figure 1:**
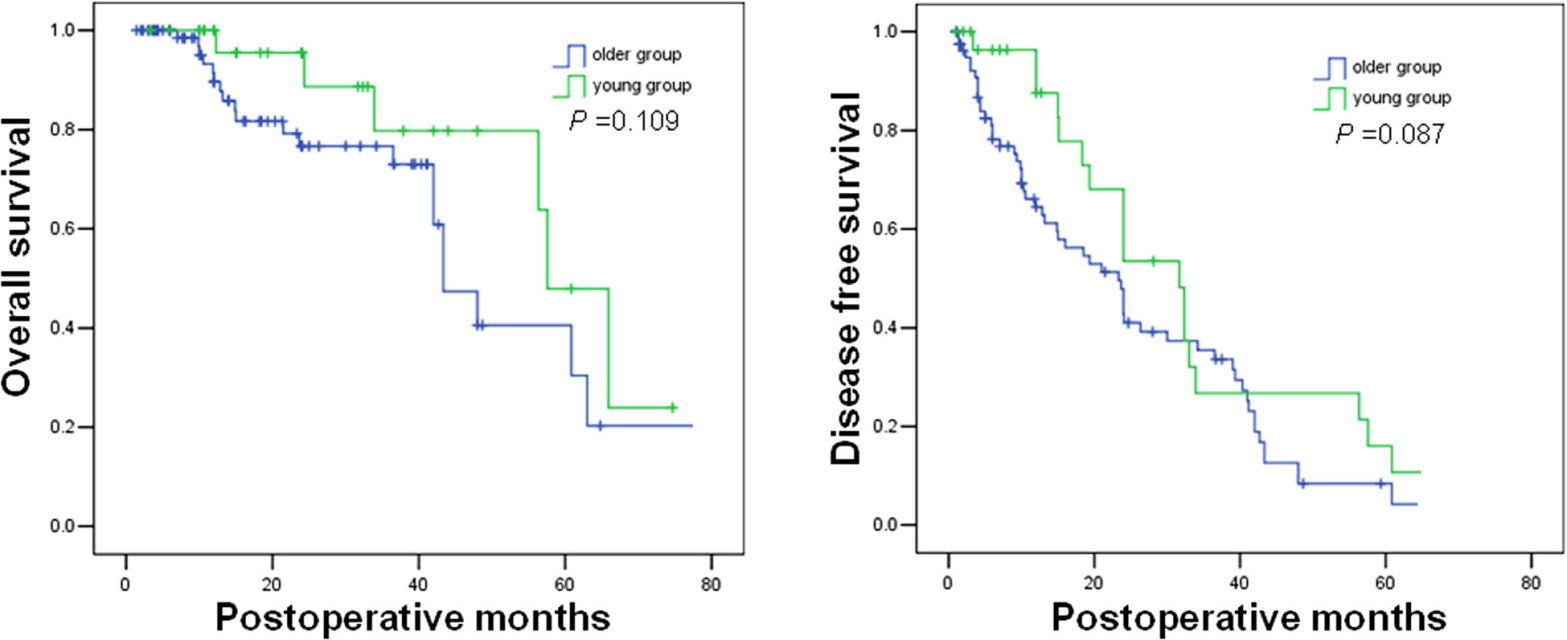
Cumulative overall and disease-free survival curves after hepatectomy of the young and older patients with large hepatocellular carcinoma.

The 1-, 3-, and 5-year overall survival rates in the young group were 93%, 79% and 47%. The 1-, 3-, and 5-year disease-free survival rates in the young group were 87%, 28% and 17%. The 1-, 3-, and 5-year overall survival rates in the older group were 85%, 75% and 40%. The 1-, 3-, and 5-year disease-free survival rates in the older group were 65%, 36% and 11%. Overall survival and disease-free survival in the young group and the older group were similar (*P* = 0.109 and *P* = 0.087, respectively; Figure 1).

Variables that might affect overall survival of young patients with large HCC after hepatic resection were also analyzed in this study (Table 4). Univariate analysis of the prognostic factorsfound that patients with liver cirrhosis (*P* = 0.045), vascular invasion (*P* = 0.017) and high Edmondson-Steiner grade (*P* = 0.036) had poorer overall survival than those without these variables. However, using multivariate analysis of the prognostic factors that predicted overall survival status, only presence of vascular invasion (*P* = 0.031) and high Edmondson-Steiner grade (*P* = 0.042) was significant (Table 4).

**Table 4:** Cox proportional hazard regression analyses for overall survival in young patients with large hepatocellular carcinoma after hepatectomy

**Variables**	n*	**Univariateanalysis**		**Multivariate analysis**	
		**HR (95% CI)**	***P *** **value**	**HR (95% CI)**	***P *** **value**
Gender					
Female	17	1		1	
Male	53	0.752(0.290-3.547)	0.548	0.721(0.309-3.176)	0.408
Hepatitis B status					
Positive	61	1		1	
Negative	9	0.963(0.243-4.416)	0.363	0.904(0.262-3.455)	0.789
AFP level (ng/mL)					
Negative	21	1		1	
Positive	49	1.006(0.561-1.012)	0.053	1.069(0.972-1.108)	0.129
Number of tumors					
Single	49	1		1	
Multiple	21	1.351(0.739-2.146)	0.053	1.683(0.460-2.373)	0.112
Liver cirrhosis					
Absent	38	1		1	
Present	32	1.114(1.058-2.934)	**0.045**	1.137(0.866-2.798)	0.207
Child-Pugh classification					
A	56	1		1	
B	14	1.237(0.762-2.387)	0.508	1.119(0.428-3.946)	0.623
Vascular invasion					
Absent	37	1		1	
Present	33	2.112(1.037-3.896)	**0.017**	2.233(1.010-4.232)	**0.031**
Edmondson-Steiner grade					
Low grade (I and II)	34	1		1	
High grade (III and IV)	36	1.560(1.087-3.331)	**0.036**	1.747(1.235-3.346)	**0.042**
Surgical resection margin					
≤1 cm	34	1		1	
>1 cm	36	1.339(0.234-3.642)	0.743	1.366(0.356-2.956)	0.375
Blood loss (ml)					
<1,000	36	1		1	
≥1,000	34	1.036(0.424-2.986)	0.117	1.352(0.374-3.463)	0.353
Blood transfusion (ml)					
Without	32	1		1	
With	38	1.008(0.532-1.787)	0.733	1.453(0.834-2.564)	0.656
Complications					
Absent	54	1		1	
Present	16	0.986(0.330-2.675)	0.559	0.824(0.363-2.863)	0.348
BMI					
Normal weight	55	1		1	
Overweight/obese	15	1.006(0.687-1.331)	0.309	1.250(0.909-1.671)	0.326

*Number of patients. *P* values shown in bold indicate statistical significance between groups. AFP, alpha-fetoprotein; BMI, body mass index; HR, hazard ratio.

## Discussion

Young patients with large HCC, who are rare in USA and Europe, are not uncommon at diagnosis in China. Astudy showed that 30% of HCC patients were younger than 40 years old [8]; in our cohort this ratio reached 47%. The high liver cancer rates in young patients in China largely reflect the prevalence of chronic hepatitis B virus (HBV) infection [9-11]. In this study, the young group had more HBV-related HCC than the older group (87.2% vs66.3%, *P* = 0.031). This implied that HCC in most young patients was caused by HBV infection. This infection may even have happened in infancy and has caused liver cirrhosis after many years. HBV infection leads to HCC [12-15]; therefore, regular examination of AFP and B ultrasound is very important for young patients with a history of hepatitis B infection. It is key for an early diagnosis and early operative treatment to improve the survival rate of young HCC patients. In the older group, there may be other factors involved. In the young group, 15 patients (21.4%) were overweight (BMI 25 to 29.9 kg/m^2^) or obese (BMI ≥30 kg/m^2^), compared to 38 patients (45.8%) in the older group (*P* = 0.032). It is implied that non-alcoholic fatty liver diseases, which are associated with obesity, may participate in the development of HCC, especially in older patients [16-19]; therefore, we must pay more attention to the older patients who are overweight or obese.

Our study showed that hepatic resection for large HCC could be performed with an acceptable mortality rate and postoperative complication rates. The perioperative data were similar between two two groups, except that the older group had more pulmonary infection after hepatectomy. This implies that hepatectomy for young patients with large HCC is safe. We also found that the cumulative overall and disease-free survival curves after hepatectomy showed no statistical difference between the young and the older groups. This implies that hepatic resection for young patients with large HCC is efficacious.

## Conclusion

In conclusion, the clinicopathologic characteristics and the outcome for young patients with large HCC after liver resection were similar to that of the older patients, except for differences in infection through HBV and BMI. Liver resection for young patients with large HCC is safe and efficacious and should be recommended.

## Abbreviations

AFP: alpha-fetoprotein; BMI: body mass index; HBV: hepatitis B virus; HCC: hepatocellular carcinoma.

## Competing interests

The authors declare that they have no competing interests.

## Authors’ contributions

XYL and JFX carried out the data collection and analysis, drafted the manuscript. Both authors read and approved the final manuscript.
